# Correction: The interface between SARS-CoV-2 and non-communicable diseases (NCDs) in a high HIV/TB burden district level hospital setting, Cape Town, South Africa

**DOI:** 10.1371/journal.pone.0324096

**Published:** 2025-06-06

**Authors:** The PLOS One Staff

The Competing interests section for this article is incorrect. The correct Competing interests section is as follows: The authors declare no competing interests.

The publisher apologizes for the error.
