# Supplementary material for: The interface between SARS-CoV-2 and non-communicable diseases (NCDs) in a high HIV/TB burden district level hospital setting, Cape Town, South Africa
Source: PLoS One. 2023 Oct 5;18(10):e0277995. doi: 10.1371/journal.pone.0277995 (PMC10553288; doi:10.1371/journal.pone.0277995)
Supplement: Attachment — Submitted filename: PLOS one review_30.11.22.docx [file pone.0277995.s002.docx]

PLOS one review

Abstract:

Introduction

Line 52: For the sake of consistency, I suggest you use 13.7% instead of 13,7%.

Methods

Line 108: …..admission, from digital registries, with additional data were captured from electronic…

Comment: …with additional data captured from. Delete ‘were’.

Line 113: 30 kg/m2

Comment: It is square, and not 2 (kg/m^2^)

Line 126: ….and standard deviation (SE)

Comment: Do you mean standard deviation (SD)?

Results

Line 150: Table 1

Comment: I think the ‘N’ column is not necessary. Apart from HIV/AIDS, the rest are 560. I suggest you put asterisk (*) on the HIV/AIDS variable, and define/explain the denominator below the table.

Age class (years)

On line 82 (study population), you mentioned individuals aged 18 years and above were recruited. Why do you have an age group/class from 14 years?? If you think it’s from 14 years, kindly correct that in the study population.

References

Line 435:

Comment: All your on-line references must include dates accessed.

Additional comments:

- Is Figure 1 an original map generated by the authors? If no, kindly reference the source.
- The title for figure 1 needs to be enhanced. ‘Khayelitsha District in Cape Town/South Africa’.
- I suggest ‘Map of xxx showing xxx District. And please do not combine Cape Town/South Africa like that. I guess they are not the same, so please be specific. If the map is a South African map, say so; if it is a map of Cape Town, let the readers know.
- Again, kindly delete ‘Map of Cape Town/ Khayelitsha District’ on the top left corner of the map.
