# Supplementary material for: The interface between SARS-CoV-2 and non-communicable diseases (NCDs) in a high HIV/TB burden district level hospital setting, Cape Town, South Africa
Source: PLoS One. 2023 Oct 5;18(10):e0277995. doi: 10.1371/journal.pone.0277995 (PMC10553288; doi:10.1371/journal.pone.0277995)
Supplement: Attachment — Submitted filename: Reviewers_responses_08_Dec_2022.docx [file pone.0277995.s003.docx]

**Reviewers’ responses**

Manuscript: **The interface between SARS-CoV-2 and non-communicable diseases (NCDs) in a high HIV/TB burden district level hospital setting, Cape Town, South Africa**

**Editor**

**Comment 1:**  Please ensure that your manuscript meets PLOS ONE's style requirements, including those for file naming. The PLOS ONE style templates can be found at

[https://journals.plos.org/plosone/s/file?id=wjVg/PLOSOne_formatting_sample_main_body.pdf](https://eur03.safelinks.protection.outlook.com/?url=https%3A%2F%2Fjournals.plos.org%2Fplosone%2Fs%2Ffile%3Fid%3DwjVg%2FPLOSOne_formatting_sample_main_body.pdf&data=05%7C01%7C%7Cb0cedeab351c4b58f8cf08dad47189a5%7Ca6fa3b030a3c42588433a120dffcd348%7C0%7C0%7C638055882029843275%7CUnknown%7CTWFpbGZsb3d8eyJWIjoiMC4wLjAwMDAiLCJQIjoiV2luMzIiLCJBTiI6Ik1haWwiLCJXVCI6Mn0%3D%7C3000%7C%7C%7C&sdata=7EZup2qczCr%2FifaCKkLIaPNGGdTLGv5gVqgB9Crz0Ww%3D&reserved=0)  and

[https://journals.plos.org/plosone/s/file?id=ba62/PLOSOne_formatting_sample_title_authors_affiliations.pdf](https://eur03.safelinks.protection.outlook.com/?url=https%3A%2F%2Fjournals.plos.org%2Fplosone%2Fs%2Ffile%3Fid%3Dba62%2FPLOSOne_formatting_sample_title_authors_affiliations.pdf&data=05%7C01%7C%7Cb0cedeab351c4b58f8cf08dad47189a5%7Ca6fa3b030a3c42588433a120dffcd348%7C0%7C0%7C638055882029843275%7CUnknown%7CTWFpbGZsb3d8eyJWIjoiMC4wLjAwMDAiLCJQIjoiV2luMzIiLCJBTiI6Ik1haWwiLCJXVCI6Mn0%3D%7C3000%7C%7C%7C&sdata=UNivmbaN8c11ZxPBNVgGchlHqAdDxuEYmO0%2BMEktcuU%3D&reserved=0)

***Response 1:*** *Thank you for this comment. This manuscript has been written in accordance with the PLOS One guideline.*

**Comment 2:** Please provide additional details regarding participant consent. In the ethics statement in the Methods and online submission information, please ensure that you have specified (1) whether consent was informed and (2) what type you obtained (for instance, written or verbal, and if verbal, how it was documented and witnessed). If your study included minors, state whether you obtained consent from parents or guardians. If the need for consent was waived by the ethics committee, please include this information.

If you are reporting a retrospective study of medical records or archived samples, please ensure that you have discussed whether all data were fully anonymized before you accessed them and/or whether the IRB or ethics committee waived the requirement for informed consent. If patients provided informed written consent to have data from their medical records used in research, please include this information.

***Response 2:*** *Thanks. In the tracking manuscript, line 142-145, we have stated the following: “Since the data were obtained from hospital records, a waiver of consent was requested and granted. The data were entered and analyzed anonymously, and no attempt was made to link the data to an individual identifier”.*

**Comment 3:** Thank you for stating the following financial disclosure:

 “The funders had no role in study design, data collection and analysis, decision to publish, or preparation of the manuscript”

At this time, please address the following queries:

a) Please clarify the sources of funding (financial or material support) for your study. List the grants or organizations that supported your study, including funding received from your institution.

b) State what role the funders took in the study. If the funders had no role in your study, please state: “The funders had no role in study design, data collection and analysis, decision to publish, or preparation of the manuscript.”

c) If any authors received a salary from any of your funders, please state which authors and which funders.

d) If you did not receive any funding for this study, please state: “The authors received no specific funding for this work.”

Please include your amended statements within your cover letter; we will change the online submission form on your behalf.

***Response 3:*** *In the tracking manuscript 452-452,* *we have stated that “The authors received no specific funding for this work.”*

**Comment 4:** Thank you for stating the following in your Competing Interests section:

“I have read the journal's policy and the authors of this manuscript have the following competing interests”

Please complete your Competing Interests on the online submission form to state any Competing Interests. If you have no competing interests, please state "The authors have declared that no competing interests exist.", as detailed online in our guide for authors at <http://journals.plos.org/plosone/s/submit-now>

 This information should be included in your cover letter; we will change the online submission form on your behalf.

***Response 4:*** *Thanks. In the tracking manuscript line 449-450, we have stated that "The authors have declared that no competing interests exist."*

Comment 5: We note that you have stated that you will provide repository information for your data at acceptance. Should your manuscript be accepted for publication, we will hold it until you provide the relevant accession numbers or DOIs necessary to access your data. If you wish to make changes to your Data Availability statement, please describe these changes in your cover letter and we will update your Data Availability statement to reflect the information you provide.

***Response 5:*** *Thanks. In the tracking manuscript line 453-455, the data availability has been changed as follows: “The dataset can be obtained by submitting a request form to the corresponding author.*

**Comment 6**: Your ethics statement should only appear in the Methods section of your manuscript. If your ethics statement is written in any section besides the Methods, please delete it from any other section.

***Response 6:*** *Thanks. In the tracking manuscript line 445-446, we have deleted the ethics statement.*

**Comment 7**: Please include a separate caption for each figure in your manuscript.

***Response 7:*** *Thanks. We have included all the figures in the manuscript.*

**Comment 8:** We note that Figure 1 in your submission contain [map/satellite] images which may be copyrighted. All PLOS content is published under the Creative Commons Attribution License (CC BY 4.0), which means that the manuscript, images, and Supporting Information files will be freely available online, and any third party is permitted to access, download, copy, distribute, and use these materials in any way, even commercially, with proper attribution. For these reasons, we cannot publish previously copyrighted maps or satellite images created using proprietary data, such as Google software (Google Maps, Street View, and Earth). For more information, see our copyright guidelines: <http://journals.plos.org/plosone/s/licenses-and-copyright>.

We require you to either (1) present written permission from the copyright holder to publish these figures specifically under the CC BY 4.0 license, or (2) remove the figures from your submission:

a. You may seek permission from the original copyright holder of Figure 1 to publish the content specifically under the CC BY 4.0 license.

We recommend that you contact the original copyright holder with the Content Permission Form (<http://journals.plos.org/plosone/s/file?id=7c09/content-permission-form.pdf>) and the following text:

“I request permission for the open-access journal PLOS ONE to publish XXX under the Creative Commons Attribution License (CCAL) CC BY 4.0 (<http://creativecommons.org/licenses/by/4.0/>). Please be aware that this license allows unrestricted use and distribution, even commercially, by third parties. Please reply and provide explicit written permission to publish XXX under a CC BY license and complete the attached form.”

Please upload the completed Content Permission Form or other proof of granted permissions as an "Other" file with your submission.

In the figure caption of the copyrighted figure, please include the following text: “Reprinted from [ref] under a CC BY license, with permission from [name of publisher], original copyright [original copyright year].”

b. If you are unable to obtain permission from the original copyright holder to publish these figures under the CC BY 4.0 license or if the copyright holder’s requirements are incompatible with the CC BY 4.0 license, please either i) remove the figure or ii) supply a replacement figure that complies with the CC BY 4.0 license. Please check copyright information on all replacement figures and update the figure caption with source information. If applicable, please specify in the figure caption text when a figure is similar but not identical to the original image and is therefore for illustrative purposes only.

The following resources for replacing copyrighted map figures may be helpful:

USGS National Map Viewer (public domain): <http://viewer.nationalmap.gov/viewer/>

The Gateway to Astronaut Photography of Earth (public domain): <http://eol.jsc.nasa.gov/sseop/clickmap/>

Maps at the CIA (public domain): <https://www.cia.gov/library/publications/the-world-factbook/index.html> and <https://www.cia.gov/library/publications/cia-maps-publications/index.html>

NASA Earth Observatory (public domain): <http://earthobservatory.nasa.gov/>

Landsat: <http://landsat.visibleearth.nasa.gov/>

USGS EROS (Earth Resources Observatory and Science (EROS) Center) (public domain): [http://eros.usgs.gov/#](http://eros.usgs.gov/)

Natural Earth (public domain): <http://www.naturalearthdata.com/>

***Response 8:*** *Thanks, the map was created using a shapefile from the Africa map library software, which was then imported into the Quantum Geographic information system (QGIS) software for georeferencing of the Khayelitsha district.*

**Comment 9:** Please review your reference list to ensure that it is complete and correct. If you have cited papers that have been retracted, please include the rationale for doing so in the manuscript text, or remove these references and replace them with relevant current references. Any changes to the reference list should be mentioned in the rebuttal letter that accompanies your revised manuscript. If you need to cite a retracted article, indicate the article’s retracted status in the References list and also include a citation and full reference for the retraction notice.

***Response 9:*** *Thanks. In the tracking manuscript, the following references 1, 2, 3, 8, 13, 14, 15, 16, 17, 22, 23, 24, 26, 27, and 38 have been revised accordingly.*

**Reviewer 1**

**Comment 1:** Line 52: For the sake of consistency, I suggest you use 13.7% instead of 13,7%.

***Response 1:*** *Thanks. In the tracking manuscript line 52, we have corrected as follows: “HIV prevalence is 13.7%”*

**Comment 2:** Line 108: …..admission, from digital registries, with additional data were captured from electronic…Comment: …with additional data captured from. Delete ‘were’.

***Response 2:*** *Thanks. In the tracking manuscript line 111, “were” has been deleted.*

**Comment 3:** Line 113: 30 kg/m2 Comment: It is square, and not 2 (kg/m^2^)

***Response 3:*** *In tracking manuscript line 116, the change has been made as follows: “30 kg/m^2^”*

Comment 4: Line 126: ….and standard deviation (SE) Comment: Do you mean standard deviation (SD)?

***Response 4:*** *Thanks. This has been corrected in the tracking manuscript line 129.*

**Comment 5:** Line 150: Table 1. Comment: I think the ‘N’ column is not necessary. Apart from HIV/AIDS, the rest are 560. I suggest you put asterisk (*) on the HIV/AIDS variable, and define/explain the denominator below the table.

***Response 5:*** *Thanks for this valuable comment. In the tracking manuscript line 160-161 and 165 “N” column has been deleted. We have also placed (*) on HIV/AIDS and explained this below the table as follows:”* *HIV status was only available for 428 of the 560 patients”.*

**Comment 6:** Age class (years). On line 82 (study population), you mentioned individuals aged 18 years and above were recruited. Why do you have an age group/class from 14 years?? If you think it’s from 14 years, kindly correct that in the study population.

***Response 6:*** *Thanks for this important comment. Age in years has been included in table 1. The study included the adult population. In the tracking manuscript Table 1, we have corrected this.*

**Comment 7:** Line 435: Comment: All your online references must include dates accessed.

***Response 7:*** *Thanks. In the tracking manuscript, the following references 1, 2, 3, 8, 13, 14, 15, 16, 17, 22, 23, 24, 26, 27, and 38 have been revised accordingly.*

**Comment 8:** Is Figure 1 an original map generated by the authors? If no, kindly reference the source.

- The title for figure 1 needs to be enhanced. ‘Khayelitsha District in Cape Town/South Africa’.
- I suggest ‘Map of xxx showing xxx District. And please do not combine Cape Town/South Africa like that. I guess they are not the same, so please be specific. If the map is a South African map, say so; if it is a map of Cape Town, let the readers know.
- Again, kindly delete ‘Map of Cape Town/ Khayelitsha District’ on the top left corner of the map.

***Response 8:*** *Thanks, the map was created using a shapefile from the Africa map library software, which was then imported into the Quantum Geographic Information system (QGIS) software for georeferencing of the Khayelitsha district. In addition to this, the title of the map has been changed as follows: “Figure 1: Map of Cape Town showing* the geographical position of *Khayelitsha District”. We also removed the text 'Map of Cape Town/Khayelitsha District' from the map's top left corner.*
